# The spectrum of co-existing disease in children with established kidney failure using registry and linked electronic health record data

**DOI:** 10.1007/s00467-024-06470-x

**Published:** 2024-08-08

**Authors:** Lucy Plumb, Retha Steenkamp, Alexander J. Hamilton, Heather Maxwell, Carol D. Inward, Stephen D. Marks, Dorothea Nitsch

**Affiliations:** 1 UK Renal Registry, UK Kidney Association, Brandon House Building 20A1, Filton 20, Filton, Bristol, BS34 7RR UK; 2 Population Health Sciences, University of Bristol Medical School, Bristol, UK; 3 Department of Paediatric Nephrology, Royal Hospital for Children, Glasgow, UK; 4 Department of Paediatric Nephrology, University Hospitals Bristol & Weston NHS Foundation Trust, Bristol, UK; 5 Department of Paediatric Nephrology, Great Ormond Street Hospital for Children, NHS Foundation Trust, London, UK; 6 NIHR Great Ormond Street Hospital Biomedical Research Centre, University College London Great Ormond Street Institute of Child Health, London, UK; 7 Department of Non-Communicable Disease Epidemiology, London School of Hygiene and Tropical Medicine, London, UK

**Keywords:** Electronic health records, Kidney failure, Kidney replacement therapy, Paediatrics, Child, Comorbidity

## Abstract

**Background:**

Children with established kidney failure may have additional medical conditions influencing kidney care and outcomes. This cross-sectional study aimed to examine the prevalence of co-existing diseases captured in the electronic hospital record compared to UK Renal Registry (UKRR) data and differences in coding.

**Methods:**

The study population comprised children aged < 18 years receiving kidney replacement therapy (KRT) in England and Wales on 31/12/2016. Comorbidity data at KRT start was examined in the hospital record and compared to UKRR data. Agreement was assessed by the kappa statistic. Associations between patient and clinical factors and likelihood of coding were examined using multivariable logistic regression.

**Results:**

A total of 869 children (62.5% male) had data linkage for inclusion. UKRR records generally reported a higher prevalence of co-existing disease than electronic health records; congenital, non-kidney disease was most commonly reported across both datasets. The highest sensitivity in the hospital record was seen for congenital heart disease (odds ratio (OR) 0.65, 95% confidence interval (CI) 0.51, 0.78) and malignancy (OR 0.63, 95% CI 0.41, 0.85). At best, moderate agreement (kappa ≥ 0.41) was seen between the datasets. Factors associated with higher odds of coding in hospital records included age, while kidney disease and a higher number of comorbidities were associated with lower odds of coding.

**Conclusions:**

Health records generally under-reported co-existing disease compared to registry data with fair-moderate agreement between datasets. Electronic health records offer a non-selective overview of co-existing disease facilitating audit and research, but registry processes are still required to capture paediatric-specific variables pertinent to kidney disease.

**Graphical Abstract:**

**Figure:**
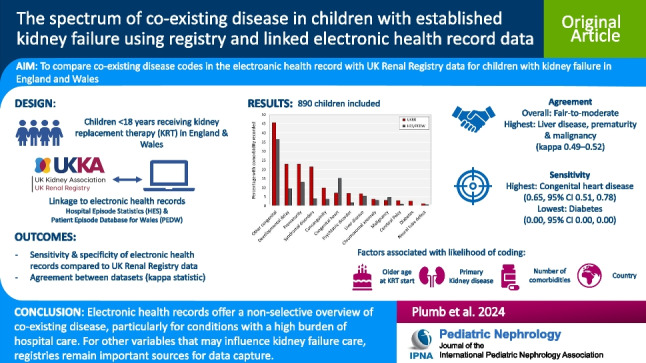
A higher resolution version of the Graphical abstract is available as Supplementary information

**Supplementary Information:**

The online version contains supplementary material available at 10.1007/s00467-024-06470-x.

## Introduction

Co-existing disease or comorbidity is common among children with established kidney failure [1]. Nationally representative data from the UK Renal Registry (UKRR) has shown that of children who commence long-term kidney replacement therapy (KRT), 16.6% have one reported comorbidity, while 9.1% have two or more [2]. Compared to those without comorbidity, studies have shown the presence of co-existing disease is an important predictor of access to kidney transplantation [3, 4], hospitalisation frequency [1] and mortality for children with kidney failure [1, 4–6]. Furthermore, the presence of comorbidity may influence treatment decisions, including whether to commence long-term KRT or opt for conservative care [7–9].

Given the influence co-existing disease has on clinically relevant outcomes for children with kidney failure, it is imperative that accurate and complete data is available to avoid selection bias and to ensure appropriate adjustment for case mix in statistical analyses [10, 11]. Efforts to increase the completeness and accuracy of comorbidity data may include merging of external datasets to supplement and validate those held by renal registries [12]. Electronic health record data offer patient-level information about care at the point of delivery and as such are representative of ‘real world’ clinical encounters [13] that are at lower risk of attrition and recall bias and are relatively inexpensive to use compared to manual data collection. Since 2018, there has been a linkage between the UKRR (kidney failure, chronic kidney disease and acute kidney injury datasets) and the electronic hospital record datasets for England (Hospital Episode Statistics, HES) and Wales (the Patient Episode Database for Wales, PEDW) for this purpose.

For children, few studies exist which examine the accuracy of diagnostic recording of chronic conditions in routinely collected health data [14, 15]. To our knowledge, no study has compared information on co-existing conditions from registry data with that held in electronic health records. The aims of this study were to therefore describe the prevalence of co-existing disease for children receiving long-term KRT as captured within the UKRR kidney failure dataset and the linked hospital record; to determine the concordance between the two datasets; and to evaluate whether there are systemic factors associated with the likelihood of coding in the electronic health record.

## Materials and methods

### Study population

A cross-sectional study was performed to address the above aims. The study population comprised children aged under 18 years receiving long-term (> 90 days) KRT from paediatric hospitals based in England and Wales at the end of 31/12/2016.

### Data sources

The UKRR collects, reports and analyses clinical data on all children in the UK receiving long-term KRT through automated data extraction from paediatric nephrology centre-specific electronic systems and clinician reporting. For children, information is collected on 13 co-existing conditions considered important and relevant to kidney care and outcomes (Table 1). These are coded as yes/no or may be missing. Co-existing disease variables are routinely considered part of the non-mandatory dataset; therefore, results that are incongruous, missing or invalid will be queried with local clinicians for each paediatric nephrology centre, but priority is given to obtaining returns for mandatory items. Due to the high proportions of missing comorbidity data, a national audit was conducted in 2017 by the UKRR with the support of the British Association for Paediatric Nephrology to improve the completeness of co-existing disease data at KRT start for children receiving long-term KRT. Clinicians from each of the 13 UK paediatric nephrology centres were asked to review the data held by the UKRR for each of their prevalent patients receiving long-term KRT at the end of 2016 and to supplement this where missing or additional information was required.

**Table 1:** Comorbidity variables at KRT start collected by the UK Renal Registry (clinicians are asked to record as yes/no/missing)

Comorbidity variable
Cerebral palsy
Congenital (non-kidney) anomaly
Congenital heart disease
Consanguinity
Chromosomal anomaly
Developmental delay
Diabetes
Liver disease
Malignancy
Neural tube defect
Prematurity
Psychiatric disorders
Syndromal disorders

Using National Health Service (NHS) number and date of birth, these data were linked to Hospital Episode Statistics (HES) and Patient Electronic Database for Wales (PEDW) datasets, the electronic records of all hospital activity occurring at NHS Hospitals in England and Wales, respectively. HES data covers the activity of all NHS hospitals in England, which also includes private patients treated at NHS hospitals, patients who are treated by private or independent (non-NHS) providers where that treatment is funded by the NHS and patients who reside outside of England but who are treated within NHS hospitals in England [16, 17]. The PEDW database is compiled from patient records generated by administrative systems of all inpatient and day case activity occurring within Welsh hospitals as well as data on Welsh residents being treated in English trusts [18]. Since 1997, PEDW datasets have sought to align data capture with that of HES to enable benchmarking of care and are broadly comparable [19]. For the purposes of this study, the admitted patient care dataset (APC) for both HES and PEDW was used to ascertain co-existing diagnoses in the electronic health record. For each clinical encounter, diagnostic codes are derived from hospital notes by clinical coders using the International Classification of Diseases 10th revision (ICD-10 from 1st April 1995). Diagnostic codes and admissions captured within the HES and PEDW APC up to the date of starting KRT were used to quantify and describe the prevalence of co-existing disease among children on KRT. Children receiving care outside of England or Wales, or those for whom linkage to HES or PEDW was not possible, were excluded from analyses.

### Comorbidity codelist development

For direct comparison with hospital record data, lists of ICD-10 codes were compiled for each corresponding UKRR-captured comorbidity variable. A systematic approach was taken to identifying relevant codes. For each comorbidity variable, relevant diagnoses and conditions for each variable were identified using data dictionaries and reviewed by an independent reviewer to determine inclusion (Supplemental Table 1). As UKRR-defined comorbidity may not fully capture all disease categories, co-existing disease was also quantified and described by ICD-10 disease chapters (Supplemental Table 2).

### Data analysis

Baseline characteristics of the study cohort were described using descriptive statistics. Continuous variables were reported using the mean (standard deviation) or median (25th–75th interquartile range (IQR)), and categorical variables as percentages. Comorbidity recording within the hospital record was compared to that captured by UKRR, which was considered the ‘gold standard’ as comorbidity data quality and completeness were improved for children receiving KRT in 2016. Sensitivity (proportion of true cases in hospital record), specificity (proportion of true ‘non cases’ in hospital record) and positive predictive value (PPV, probability that HES/PEDW-recorded comorbidity is also present in UKRR dataset) were calculated for each comorbidity. Agreement between HES/PEDW and UKRR data was assessed using the kappa statistic and 95% confidence intervals (95% CI).

To examine whether any systematic differences exist in children with and without the corresponding HES/PEDW coding for UKRR comorbidities, univariable and multivariable (age- and sex-adjusted) logistic regression was used to examine associations between demographic (e.g. age, sex, ethnicity), clinical (e.g. primary renal disease (PRD) categorised using European Renal Association groupings [20], KRT modality) and likelihood of comorbidity coding (yes/no) in electronic health records.

## Results

At the end of 2016, there were 869 children in England and Wales receiving long-term KRT who were included in this analysis. Table 2 highlights the demographic and clinical characteristics of those included and excluded from the study cohort. Of those not included (*n* = 120), the majority (*n* = 91) resided in Scotland and Northern Ireland, devolved nations where access to the electronic health record is unavailable.

**Table 2:** Demographics of included cohort

Variable	Included cohort	Not included
**Number of children**	**869**	**120**
**Male sex %**	62.5	67.5
**Median age at first nephrology review (IQR)**	0.7 (0.05–5.4)	0.5 (0.03–5.0)
**Median age at KRT start (IQR)**	5.1 (1.6–9.6)	4.5 (2.1–8.5)
**Median age at end of 2016 (IQR)**	11.9 (7.8–15.1)	11.5 (7.3–14.9)
**Age group at end of 2016 (years) %**		
0– < 2	2.3	4.2
2– < 4	6.0	6.7
4– < 8	18.1	18.3
8– < 12	23.8	25.0
12– < 16	32.2	25.0
16–18	17.6	20.8
**Ethnicity %**		
Black	4.7	1.7
South Asian	5.1	2.6
White	68.6	89.7
Other	21.7	6.0
**PKD at start of KRT %**		
Tubulointerstitial	52.0	50.9
Systemic disease	4.5	4.2
Miscellaneous	10.4	11.9
Glomerular	16.8	22.0
Familial/hereditary	16.3	11.0
**Modality at end of 2016%**		
HD	10.7	13.5
PD	10.4	11.8
Transplant	78.9	67.2
Other	10.7	7.6
**Country %**		
England	96.1	23.3
Wales	3.9	< 1.0
Northern Ireland	0.0	25.0
Scotland	0.0	50.8
**IMD %**		
Quintile 1 (least deprived)	15.7	30.7
Quintile 2	15.9	14.9
Quintile 3	17.7	15.8
Quintile 4	22.7	20.2
Quintile 5 (most deprived)	28.1	18.4
**Number of UKRR-listed comorbidities at start of KRT %**		
0	26.2	18.8
1	33.7	36.5
2	21.0	28.2
3 +	19.1	16.5

*HD*, haemodialysis; *IMD*, index of multiple deprivation; *IQR*, interquartile range; *KRT*, kidney replacement therapy; *PD*, peritoneal dialysis; *PRD*, primary kidney disease; *UKRR*, UK Renal Registry

Over half of the included cohort were male (62.5%); the majority of children included were of White ethnicity (68.6%) compared to 77.8% of the population of England and Wales [21]. The median age of the included cohort was 11.9 (IQR 7.8–15.1) years. Most children (78.9%) had a functioning transplant at the end of 2016. Just over half (52.0%) had tubulointerstitial disease recorded as their primary kidney diagnosis which predominantly corresponds to those with congenital anomalies of the kidney and urinary tract. Overall, 73.8% of included children had evidence of a UKRR-recorded comorbidity: a third of children (33.7%) had one reported comorbidity, 21.0% had 2 comorbidities and 19.1% had three or more. Prior to the national comorbidity audit, higher proportions of children on KRT were noted to have no comorbidity (74.3%) with 16.6% recorded as having one co-existing disease and 9.1% having 2 or more [22]. Children in the included cohort were more likely to reside in the most deprived areas within the UK compared to those not included (28.1 versus 18.4%); similarly, a lower proportion of the included cohort resided in the least deprived quintile (15.7% versus 30.7% of those not included).

Within the UKRR dataset, the most common co-existing disease recorded was congenital (non-kidney) anomaly, present in 45.6% of children with data. Prematurity and developmental delay at KRT start were noted in 23% of patients respectively, while syndromal disorders were seen in over a fifth of patients contributing data (21.6%). Of those coded as being born prematurely in the UKRR dataset, 46% also had developmental delay. Just over half (54%) of children with a UKRR code for syndromal disorder had evidence of developmental delay also, with 32% coded as premature. Small proportions (< 10%) of children were noted to have each of the remaining comorbid conditions, the least frequently recorded being neural tube defect, seen in 1.2% of prevalent children at KRT start.

**Fig. 1:**
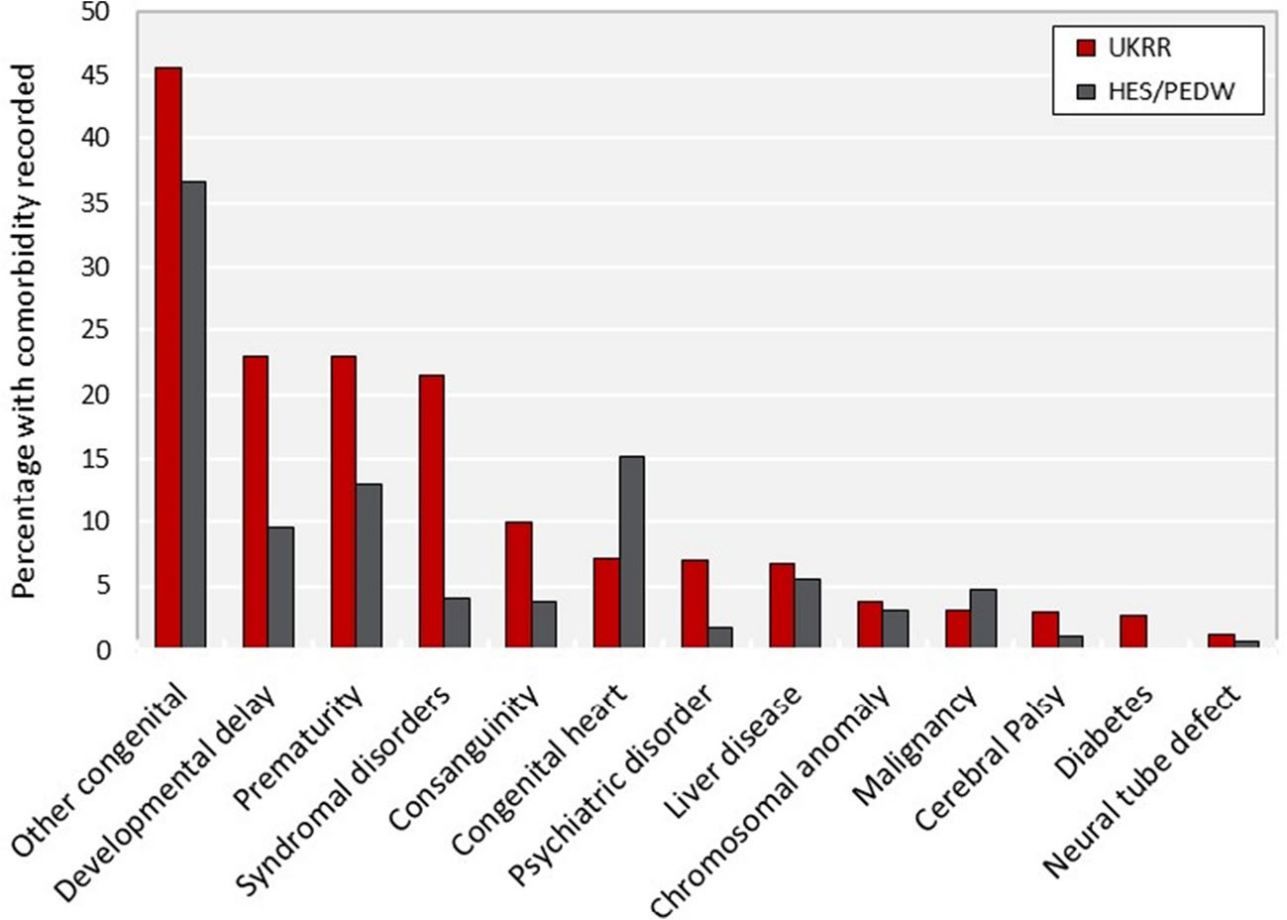
Prevalence of co-existing disease among children receiving long-term KRT, as recorded in the registry record (UKRR) and hospital dataset (HES/PEDW), respectively. Abbreviations: HES, Hospital Episodes Statistics; PEDW; Patient Episode Database for Wales; UKRR, UK Renal Registry

Overall, the UKRR records generally reported a higher prevalence of co-existing disease for most disease variables captured compared to electronic health records (Fig. 1). As with UKRR records, the comorbidity most commonly recorded in the hospital dataset was congenital (non-kidney) disease, present in 36.7% of children in the HES/PEDW datasets. For six of the 13 disease variables, broadly similar proportions of children were coded within each dataset; the largest discrepancies were noted for syndromal disorders (21.6% UKRR compared to 4% HES/PEDW) and developmental delay (23% UKRR compared to 9.6% HES/PEDW). Higher proportions of children were coded for congenital heart disease (15.2% HES/PEDW versus 7.2% UKRR) and malignancy (4.7% HES/PEDW versus 3.1% UKRR) in the electronic record compared to registry data, respectively.

**Fig. 2:**
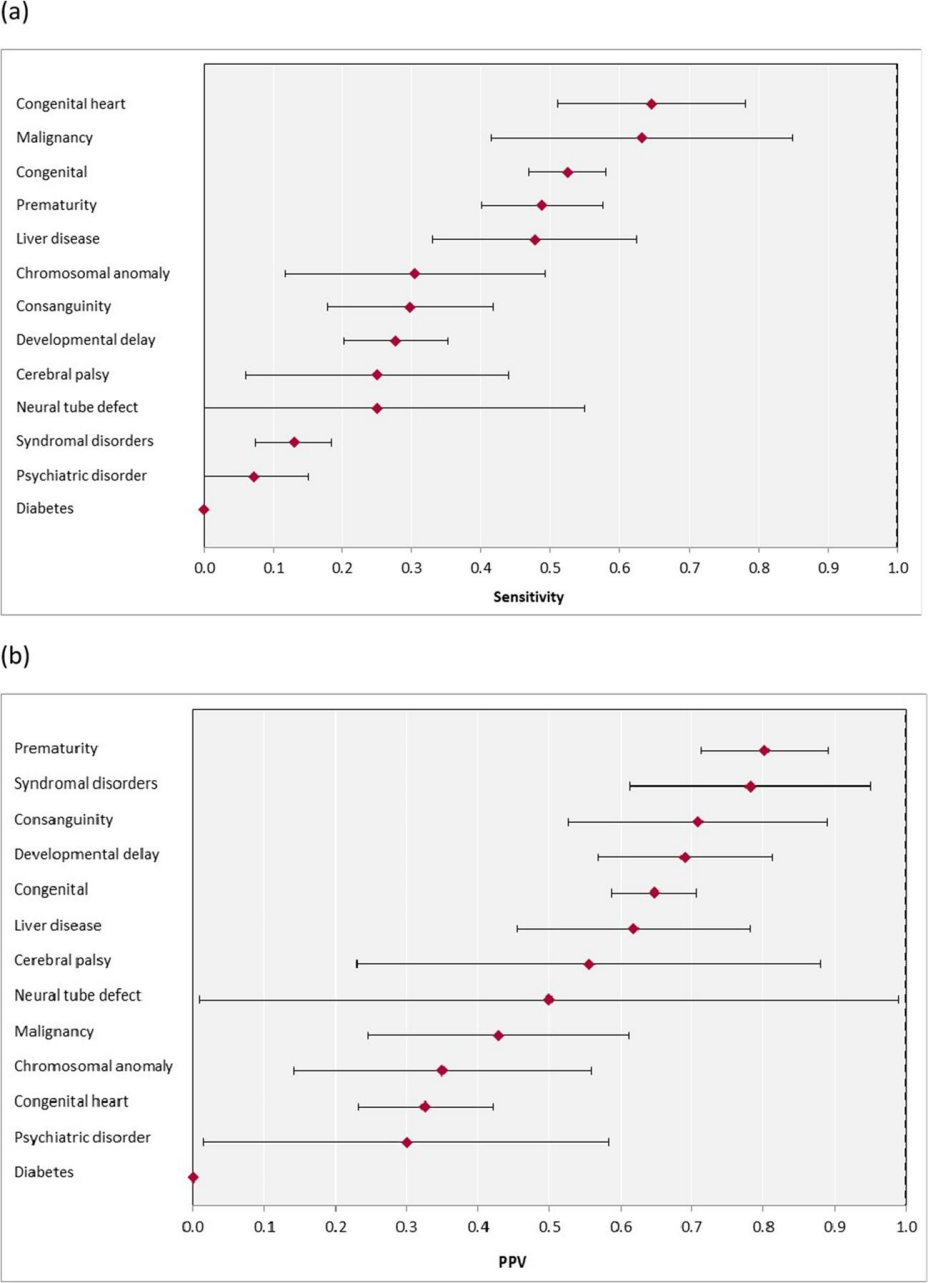
**a** Forest plot of sensitivity (%). **b** Forest plot of PPV with 95% confidence intervals for individual comorbidities derived from hospital data compared with UKRR data (gold standard). Abbreviations: *PPV*, positive predictive value; *UKRR*, UK Renal Registry

The sensitivity, specificity and PPV of each disease variable in the electronic health record were compared to the UKRR ‘gold standard’ (Table 3, Fig. 2a). The highest sensitivities were noted for congenital heart disease (0.65, 95% CI 0.51, 0.78) and malignancy (0.63, 95% CI 0.41, 0.85); the highest PPVs were noted for prematurity (0.80, 95% CI 0.71, 0.89) and syndromal disorders (PPV 0.78, 95% CI 0.61, 0.95). Highest specificities were noted for neural tube defect and diabetes (both 1.00, 95% CI 0.99, 1.00). At best, moderate agreement was seen between the two datasets (Table 3, Fig. 2b). The highest agreements were noted for prematurity (kappa statistic 0.52, 95% CI 0.43, 0.61), liver disease (kappa statistic 0.51, 95% CI 0.37, 0.65) and malignancy (kappa statistic 0.49, 95% CI 0.31, 0.67). The poorest agreements were noted for diabetes and psychiatric disorder (kappa statistic 0.01 (95% CI -0.01, 0.00) and 0.09 (95% CI -0.03, 0.21), respectively).

**Table 3:** Diagnostic utilities of hospital record comorbidity data compared to UKRR gold standard

Comorbidity	Prevalence			Kappa agreement (95% CI)	Sensitivity (95% CI)	Specificity (95% CI)
	**UKRR coding only, *N* (%)**	**HES/PEDW coding only, *N* (%)**	**Coded in both, *N* (%)**			
Cerebral palsy	15 (2.3)	5 (0.6)	5 (0.6)	0.33 (0.11–0.56)	0.25 (0.06–0.44)	0.99 (0.99–1.00)
Prematurity	67 (12.0)	49 (5.8)	61 (7.1)	0.52 (0.43–0.61)	0.49 (0.40–0.58)	0.96 (0.95–0.98)
Diabetes	18 (2.7)	2 (0.2)	0 (0.0)	0.01 (–0.01–0.00)	0.00 (0.00–0.00)	1.00 (0.99–1.00)
Developmental delay	99 (16.6)	43 (5.1)	38 (4.4)	0.30 (0.21–0.39)	0.28 (0.20–0.35)	0.96 (0.94–0.98)
Congenital	149 (22.0)	150 (17.8)	160 (18.6)	0.28 (0.21–0.36)	0.52 (0.47–0.58)	0.76 (0.71–0.80)
Congenital heart disease	17 (2.5)	97 (11.5)	31 (3.6)	0.37 (0.27–0.48)	0.65 (0.51–0.78)	0.89 (0.87–0.92)
Neural tube defect	6 (0.9)	4 (0.5)	2 (0.2)	0.33 (–0.02–0.67)	0.25 (0.00–0.55)	1.00 (0.99–1.00)
Psychiatric disorder	39 (6.5)	12 (1.4)	3 (0.4)	0.09 (–0.03–0.21)	0.07 (0.00–0.15)	0.99 (0.98–1.00)
Chromosomal anomaly	16 (2.7)	19 (2.2)	7 (0.8)	0.30 (0.11–0.49)	0.30 (0.12–0.49)	0.98 (0.96–0.99)
Liver disease	24 (3.6)	26 (3.1)	21 (2.4)	0.51 (0.37–0.65)	0.48 (0.33–0.62)	0.98 (0.97–0.99)
Consanguinity	40 (7.0)	15 (1.8)	17 (2.0)	0.38 (0.25–0.52)	0.30 (0.18–0.42)	0.99 (0.98–1.00)
Malignancy	9 (1.3)	28 (3.3)	12 (1.4)	0.49 (0.31–0.67)	0.63 (0.41–0.85)	0.97 (0.96–0.99)
Syndromal disorders	126 (18.9)	16 (1.9)	18 (2.1)	0.17 (0.10–0.25)	0.13 (0.07–0.19)	0.99 (0.98–1.00)

Prevalence percentages represent the proportion of children coded as YES for the disease variable out of those patients coded as YES or NO in the dataset. *CI*, confidence interval; *HES*, Hospital Episodes Statistics; *PEDW*; Patient Episode Database for Wales; *UKRR*, UK Renal Registry

To examine the prevalence of co-existing disease according to broader ICD-10 chapters, all co-existing diseases captured in the electronic health record are presented by sex (Supplemental Fig.  1). Genitourinary system disorders were most frequently reported, as were frequent hospital encounters (represented by health status factors, ICD-10 chapter XXI) and abnormal signs and symptoms, followed by endocrine disorders and infectious diseases. Higher proportions of male children were seen across most disease chapters, except for neoplasms, blood, circulatory and musculoskeletal disorders.

Univariable and multivariable associations with coding in the hospital record for corresponding UKRR-captured co-existing diseases are shown in Table 4. In both crude and age- and sex-adjusted analyses, older age at KRT (12– < 16 and 16– < 18 years, odds ratios (OR) 2.79 (95% CI 1.12, 6.99) and 3.66 (95% CI 1.42, 9.45), respectively) was associated with higher odds of being coded for the UKRR-captured disease variable in the hospital record, compared to 2– < 4-year-olds. A lower likelihood of coding was noted for children of Other ethnicity (compared to White, OR 0.58, 95% CI 0.37, 0.91) and those with a familial or hereditary disease (OR 0.28, 95% CI 0.15, 0.53) or tubulointerstitial primary kidney disease (0.60, 95% CI 0.38, 0.94) compared to glomerular disorders. Children receiving care in Wales had higher odds of being coded for UKRR-captured co-existing disease compared to those in England (OR 2.27, 95% CI 1.08, 4.78). Compared to children with one co-existing disease at KRT start, those with higher numbers of UKRR-defined comorbidity had increasingly lower odds of being coded as such in the hospital record.

**Table 4:** Comparison of patient and clinical characteristics for patients with and without corresponding HES/PEDW coding of UKRR-listed comorbidities

Characteristic	OR of coding in HES/PEDW for corresponding UKRR comorbidity	
	**Univariable OR (95% CI)**	**Age/sex-adjusted OR (95% CI)**
**Male sex**	0.73 (0.52, 1.03)	0.77 (0.55, 1.09)
**Age group at KRT start**		
0– < 2	3.56 (0.49, 26.02)	3.61 (0.49, 26.46)
2– < 4	1.00	1.00
4– < 8	1.72 (0.66, 4.51)	1.70 (0.65, 4.46)
8– < 12	1.72 (0.67, 4.41)	1.65 (0.64, 4.23)
12– < 16	2.87 (1.15, 7.17)	2.79 (1.12, 6.99)
16–18	3.84 (1.49, 9.90)	3.66 (1.42, 9.45)
**Ethnicity**		
Black	1.07 (0.44, 2.58)	1.00 (0.41, 2.45)
South Asian	0.57 (0.23, 1.45)	0.57 (0.22, 1.46)
White	1.00	1.00
Other	0.57 (0.37, 0.89)	0.58 (0.37, 0.91)
**PKD**		
Tubulointerstitial	0.54 (0.35, 0.84)	0.60 (0.38, 0.94)
Systemic disease	0.67 (0.29, 1.55)	0.69 (0.29, 1.63)
Miscellaneous	0.78 (0.42, 1.46)	0.80 (0.42, 1.52)
Glomerular	1.00	1.00
Familial/hereditary	0.30 (0.16, 0.56)	0.28 (0.15, 0.53)
**KRT modality at end of 2016**		
HD	1.00	1.00
PD	1.56 (0.66, 3.68)	2.05 (0.84, 4.98)
Transplant	1.30 (0.69, 2.44)	1.14 (0.59, 2.18)
**Country**		
England	1.00	1.00
Wales	2.37 (1.14, 4.95)	2.27 (1.08, 4.78)
**IMD**		
Quintile 1 (least deprived)	1.00	1.00
Quintile 2	0.69 (0.38, 1.25)	0.70 (0.38, 1.27)
Quintile 3	1.06 (0.61, 1.85)	1.09 (0.62, 1.93)
Quintile 4	1.22 (0.72, 2.06)	1.17 (0.69, 2.00)
Quintile 5 (most deprived)	0.79 (0.47, 1.34)	0.77 (0.45, 1.31)
**Number UKRR-listed comorbidities at start of KRT**		
1	1.00	1.00
2	0.30 (0.15, 0.58)	0.29 (0.15, 0.58)
3 +	0.03 (0.00, 0.18)	0.03 (0.00, 0.18)

*HES*, Hospital Episodes Statistics; *HD*, haemodialysis; *IMD*, index of multiple deprivation; *IQR*, interquartile range; *KRT*, kidney replacement therapy; *PEDW*; Patient Episode Database for Wales; *PD*, peritoneal dialysis; *PKD*, primary kidney disease; *UKRR*, UK Renal Registry

## Discussion

This study describes the burden of co-existing disease according to both registry and hospital electronic records and was able to examine the accuracy of coding of hospital records compared to the ‘gold standard’ registry reporting for 13 disease variables. It found that for children receiving long-term KRT in England and Wales, these co-existing conditions are common, with most children having at least one reported comorbidity and one in five having three or more. Overall, audited UKRR records recorded a higher prevalence of co-existing disease at KRT start; hospital records tended to under-report comorbidities. Agreement between the two datasets was generally fair, with at best moderate agreement for the captured disease variables within the registry data. We identified that the likelihood of UKRR-captured disease data was influenced by several patient and disease variables: older age at KRT start was associated with higher odds of coding in the hospital record, while being of non-White, Asian or Black ethnicity and having a familial or hereditary disorder were negatively associated with disease coding.

For children, very few data exist on the burden and type of co-existing disease seen among children with kidney failure. As with other disease-specific registries, these data are often not considered part of the ‘core’ dataset required from hospitals and are therefore often of variable completeness and quality. In an analysis of data from the International Pediatric Peritoneal Dialysis Network (IPPN) registry, up to one-third of children receiving chronic peritoneal dialysis had at least one comorbidity [1]. In comparison, we noted a higher proportion of children who had at least one comorbidity at KRT start (73.8% versus 32.9%) [1]; this difference may be due to the inclusion of children who received haemodialysis and transplantation at KRT start or differences in reporting of comorbidity, which is known to vary significantly across national registries [23]. While a similar methodology to record comorbidity was used by the UKRR and IPPN registries, a collaborative effort across UK nephrology centres to improve comorbidity records prior to this study may have helped augment data completeness, therefore resulting in a higher burden seen. The nature and distribution of co-existing disease seen however were similar, with congenital anomaly, developmental impairments and congenital heart disease commonly seen among the IPPN cohort, with frequencies broadly mirroring those observed in our study [1].

We noted general under-reporting and at best fair-to-moderate agreement for comorbidity captured in the hospital record compared to the UKRR dataset. Although few paediatric studies exist, our findings are generally consistent with previous adult reports, with low sensitivity [24–26] but high specificity [26] observed. It is likely that coding accuracy is influenced by the nature and severity of the condition and consequently its requirement for inpatient care. The finding that hospital records had relatively high sensitivity for congenital heart disease and malignancy may reflect the high burden of hospitalisation and/or admitted procedures experienced by these patients, which results in a higher likelihood of being coded in the electronic record; it is not clear however why many of the children with these conditions were not identified by the registry dataset, which warrants further investigation. Conversely, diabetes mellitus, psychiatric disorders and syndromal conditions, for which we observed lower sensitivities, may be managed predominantly in an outpatient setting and therefore less likely to be captured and coded during a hospital admission. For UK-based adult patients with chronic kidney disease, a similar pattern has been described, with diagnostic codes likely to warrant hospital admission being coded including cerebrovascular disease and ischaemic heart disease, while hypertension, dementia and smoking status were less well recorded [27]. Diagnostic certainty and ease of coding may also play a role in discrepancies observed. We noted relatively higher agreement between datasets for conditions which perhaps are likely to be easily confirmed (e.g. prematurity or malignancy) and have clear diagnostic categories within the ICD-10 chapters. From adult studies, it is also recognised that coding for chronic co-existing conditions such as chronic kidney disease may reflect more severe cases identified [28]; as the registry collects data on a yes/no/missing basis, we were unable to explore this hypothesis in greater detail.

Older age at KRT start was associated with a higher likelihood of coding for comorbidity in the electronic health record. This is perhaps unsurprising as co-existing disease was identified looking back at diagnostic fields prior to KRT start, although advancing age is also associated with higher numbers of co-existing diseases in adults [29]. Other studies have identified time as an important confounder of comorbidity reporting, with increased comorbidity yield based on the duration of data capture [25]. To counter this, it has been suggested a pre-specified timeframe (e.g. 5 years) should be employed to minimise this effect in adults [27, 30]; however, in children, this could introduce selection bias or preclude the youngest children, who are known to contribute a large proportion of the kidney failure cohort and may experience co-existing congenital disease, from inclusion [1]. Negative associations with comorbidity coding in the electronic health record included having familial/hereditary or tubulointerstitial kidney disease, being of ‘Other’ (non-White, Black or Asian) ethnicity and having a higher burden of comorbidity. Adult data have shown that higher numbers of comorbidity are associated with under-reporting in the hospital record, which is presumed to reflect a disregard for minor disease or conditions perceived to be insignificant [29]. An important finding was that children receiving kidney care in NHS hospitals in England were less likely to be coded for their UKRR-defined comorbidities compared to their Welsh counterparts. Understanding differences in clinical coding processes between the two nations may help drive quality improvement initiatives to increase coding accuracy, therefore increasing the internal validity of health data research. Although children of ‘Other’ ethnicity have historically had lower rates of hospital attendance compared to children of White, Black or Asian ethnicity, recent HES trends suggest hospital attendance rates are higher in this group [31]; therefore, the possibility of racial bias in coding cannot be discounted.

Routinely collected data must be of sufficient quality for reliable use in audit and research [32]. Overall, our findings are comparable to adult studies, highlighting that comorbidity is more likely to be under-reported in the administrative hospital record compared to other medical sources [27]; furthermore, diagnostic accuracy is lacking compared to primary diagnosis coding [32]. Our findings suggest that electronic health records may be useful to supplement data on conditions requiring hospitalisation, but for other variables that may influence the care or outcomes of a child with kidney failure, such as cognitive impairments or mental health disorders, registries remain the most appropriate resource for data capture. To that effect, it is imperative that registries continue with their efforts to collect high-quality information on co-existing disease and to prioritise comorbidity as a core variable for case-mix adjustment, given its influence on disease course and outcomes. Adult registry data analyses have suggested that patients with missing comorbidity have survival outcomes similar to multimorbid patients, thus suggesting that registry data missingness may be biased towards those with a high prevalence of co-existing disease [33], thus reinforcing the need to capture these data for meaningful analyses.

This study has a number of strengths. To our knowledge, it is the first to describe and compare the spectrum of co-existing disease for UK children with established kidney failure using linked registry and electronic health data in a nationally representative cohort of children receiving KRT. Several limitations must however be acknowledged. Our ability to directly validate co-existing disease data from the hospital record was limited to a number of pre-specified comorbidity variables collected by the UKRR, meaning the accuracy of other conditions such as respiratory disease could not be assessed. The lack of clear definitions for UKRR-captured disease variables could also leave categories open to interpretation, resulting in over-coding and misclassification of disease. This study has analysed data from NHS secondary care records; currently, the UKRR does not link to primary care data which may provide more insight into common, more granular comorbidities such as atopy. Furthermore, co-existing disease captured from private healthcare will not be captured, although for UK children, this will represent a very small proportion of medical encounters. Finally, use of linked hospital record data has enabled us to evaluate the spectrum of co-existing disease more broadly in this cohort, although it is not clear from this work whether the comorbidity identified truly represents independent extra-renal disease.

In conclusion, a higher prevalence of co-existing disease was recorded for children in England and Wales receiving long-term KRT in the audited UKRR dataset compared to the electronic health record. At best, electronic health records show moderate agreement with UKRR records for most disease variables collected. We suggest that electronic health record data can offer a non-selective overview of co-existing disease in this cohort that supports validation of routinely collected registry data and facilitates research relating to hospital encounters for co-existing disease, but that several paediatric-specific variables which may be pertinent to kidney disease management and outcomes require ongoing capture through registry processes.

## Supplementary Information

Below is the link to the electronic supplementary material.

Graphical abstract (PPTX 414 KB)

Supplementary file1 (DOCX 115 KB)

## Data Availability

The data underlying this article were provided by the UK Renal Registry and NHS Digital under license/by permission. Data will be shared on request with permission of the UK Renal Registry and NHS Digital.
