# Supplementary material for: The spectrum of co-existing disease in children with established kidney failure using registry and linked electronic health record data
Source: Pediatr Nephrol. 2024 Aug 8;39(12):3521–31. doi: 10.1007/s00467-024-06470-x (PMC11511698; doi:10.1007/s00467-024-06470-x)
Supplement: Supplementary file 1 — Graphical abstract (PPTX 414 KB) [file 467_2024_6470_MOESM1_ESM.pptx]

## Slide 1
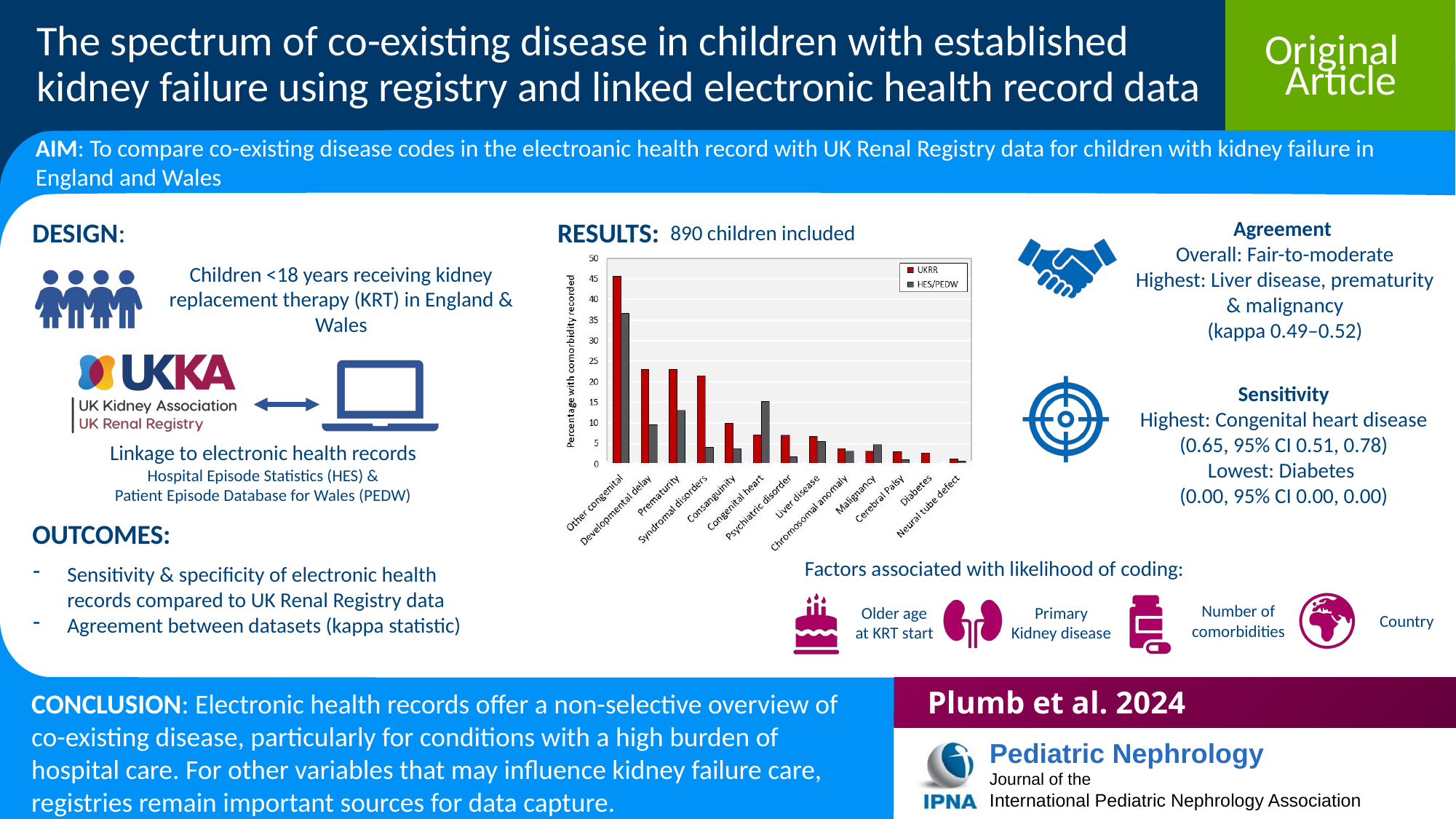

The spectrum of co-existing disease in children with established kidney failure using registry and linked electronic health record data
AIM: To compare co-existing disease codes in the electroanic health record with UK Renal Registry data for children with kidney failure in England and Wales
Agreement
Overall: Fair-to-moderate
Highest: Liver disease, prematurity & malignancy
(kappa 0.49–0.52)
DESIGN:
RESULTS:
890 children included
Children <18 years receiving kidney replacement therapy (KRT) in England & Wales
Sensitivity
Highest: Congenital heart disease (0.65, 95% CI 0.51, 0.78)
Lowest: Diabetes
(0.00, 95% CI 0.00, 0.00)
Linkage to electronic health records
Hospital Episode Statistics (HES) &
Patient Episode Database for Wales (PEDW)
OUTCOMES:
Factors associated with likelihood of coding:
Sensitivity & specificity of electronic health records compared to UK Renal Registry data
Agreement between datasets (kappa statistic)
Number of comorbidities
Older age at KRT start
Primary Kidney disease
Country
Plumb et al. 2024
CONCLUSION: Electronic health records offer a non-selective overview of co-existing disease, particularly for conditions with a high burden of hospital care. For other variables that may influence kidney failure care, registries remain important sources for data capture.
