# Supplementary material for: The spectrum of co-existing disease in children with established kidney failure using registry and linked electronic health record data
Source: Pediatr Nephrol. 2024 Aug 8;39(12):3521–31. doi: 10.1007/s00467-024-06470-x (PMC11511698; doi:10.1007/s00467-024-06470-x)
Supplement: Supplementary file 2 — Supplementary file1 (DOCX 115 KB) [file 467_2024_6470_MOESM2_ESM.docx]

Supplemental data

**The spectrum of co-existing disease in children with established kidney failure using registry and linked electronic health record data.**

Lucy Plumb^1,2^, Retha Steenkamp^1^, Alexander J Hamilton^2^, Heather Maxwell^3^, Carol D Inward^4^, Stephen D Marks^5,6^, Dorothea Nitsch^1,7^

^1^UK Renal Registry, UK Kidney Association, Bristol, UK.

^2^Population Health Sciences, University of Bristol Medical School, Bristol, UK.

^3^Department of Paediatric Nephrology, Royal Hospital for Children, Glasgow, UK

^4^Department of Paediatric Nephrology, University Hospitals Bristol & Weston NHS Foundation Trust, Bristol, UK

^5^Department of Paediatric Nephrology, Great Ormond Street Hospital for Children NHS Foundation Trust, London, UK

^6^NIHR Great Ormond Street Hospital Biomedical Research Centre, University College London Great Ormond Street Institute of Child Health, London, UK

^7^Department of Non-Communicable Disease Epidemiology, London School of Hygiene and Tropical Medicine, London, UK

Contents

[Supplemental table 1: ICD-10 codes used for deriving prevalence of UKRR comorbidity definitions 2](#_Toc164774389)

[Supplemental table 2: ICD-10 codes used for deriving prevalence of co-existing disease as per broader ICD-10 chapters 3](#_Toc164774390)

[Supplemental figure 1: Prevalence of co-existing disease from electronic health records (Hospital Episode Statistics and Patient Episode Database for Wales) by sex and ICD-10 chapter. 5](#_Toc164774391)

##

## Supplemental table 1: ICD-10 codes used for deriving prevalence of UKRR comorbidity definitions

| **UKRR comorbidities** | **Comorbidity term** | **ICD-10 codes used to derive** |
| --- | --- | --- |
| Liver disease | Diseases of the liver | K70-K77, E830-E831, G937, B15-B19, |
| Prematurity | Prematurity | P072-P073, H351 |
| Cerebral Palsy | Cerebral Palsy | G80 |
| Developmental or educational handicap | Developmental delay | F70-F73, F78-F82, F84, F88-F89, R620, R625 |
| Congenital heart disease | Congenital heart | Q20-Q26 |
| Other major congenital conditions | Other congenital | Q02-Q04, Q06, Q071-Q079, Q10-Q18, Q27-Q28, Q30-Q45, Q50-Q56, Q65-Q75, Q761-Q769, Q80-Q85, Q88-Q89 |
| Other chromasomal anomalies | Chromosomal anomaly | Q91-Q93, Q95-Q99 |
| Neural tube defect | Neural tube defect | Q000, Q01, Q05, Q070, Q760 |
| Diabetes | Diabetes | E08-E11, E13, P702 |
| Consanguinity | Consanguinity | Z843 |
| Malignancy | Malignancy | C, D0-D4 |
| Psychiatric disorder | Psychiatric disorder | F0-F6 |
| Syndromal disorders | Syndromal disorders | Q86-Q87 |

## Supplemental table 2: ICD-10 codes used for deriving prevalence of co-existing disease as per broader ICD-10 chapters

| **ICD-10 chapter** | **Comorbidity** | **ICD-10 code** |
| --- | --- | --- |
| I. Certain infectious and parasitic diseases | Infectious diseases | A, B |
| II. Neoplasms | Neoplasms | C, D1-D4 |
| III. Diseases of blood and blood-forming organs | Blood diseases | D5-D9 |
| IV. Endocrine, nutritional and metabolic diseases | Endocrine diseases | E |
| V. Mental/behavioural disorders | Mental and behavioural disorders | F |
| VI. Diseases of the nervous system | Nervous system diseases | G |
| VII. Diseases of the eye and adnexa | Eye and adenexa diseases | H0-H5 |
| VIII. Diseases of the ear and mastoid process | Ear and mastoid process diseases | H6-H9 |
| IX. Diseases of the circulatory system | Circulatory system diseases | I |
| X. Diseases of the respiratory system | Respiratory diseases | J |
| X1. Diseases of the digestive system | Digestive system diseases | K |
| XII. Diseases of skin and subcutaneous tissue | Skin diseases | L |
| XIII. Diseases of the musculoskeletal system and connective tissue | Musculoskeletal diseases | M |
| XIV. Diseases of the genitourinary system | Genitourinary system diseases | N |
| XV. Pregnancy, childbirth and the puerperium | Pregnancy and childbirth | O |
| XVI. Certain conditions originating in the perinatal period | Perinatal conditions | P |
| XVII. Congenital malformations, deformations and chromosomal abnormalities | Congenital abnormalities | Q0-Q5, Q65-Q69, Q7-Q9 |
| XVIII. Symptoms, signs and abnormal clinical and laboratory findings, not elsewhere classified | Abnormal symptoms and signs | R |
| XIX. Injury, poisoning and certain other consequences of external causes | Injury and poisoning | S, T |
| XX. External causes of morbidity and mortality | External causes | V,W, X, Y |
| XXI. Factors influencing health status and contact with health services | Health status factors | Z |
| XXII. Codes for special purposes | Special purposes | U |

##
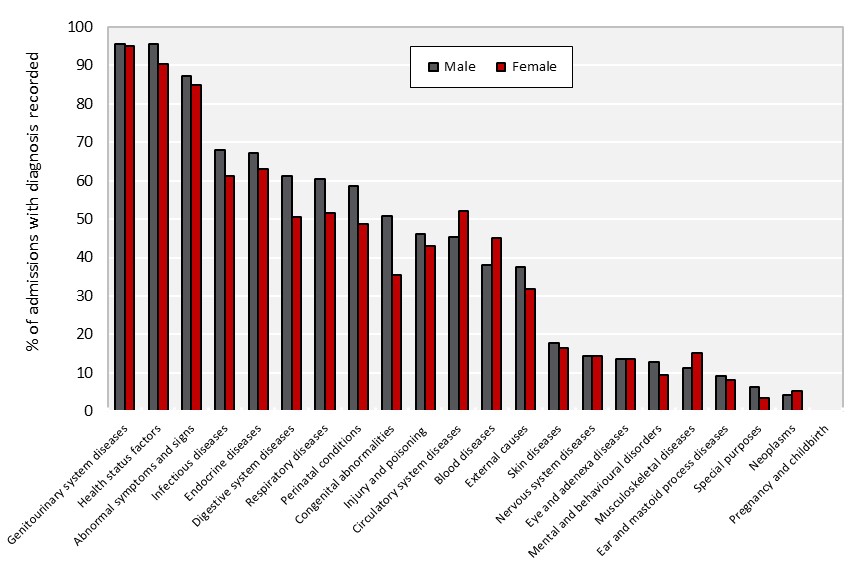
Supplemental figure 1: Prevalence of co-existing disease from electronic health records (Hospital Episode Statistics and Patient Episode Database for Wales) by sex and ICD-10 chapter.
